# Lay etiology concepts of cancer patients do not correlate with their usage of complementary and/or alternative medicine

**DOI:** 10.1007/s00432-022-04528-7

**Published:** 2023-01-18

**Authors:** J. Huebner, R. Muecke, O. Micke, F.-J. Prott, L. Josfeld, J. Büntzel, J. Büntzel, the Working Group Prevention, Integrative Oncology of the German Cancer Society

**Affiliations:** 1 Klinik Für Innere Medizin II, Universitätsklinikum Jena, Am Klinikum 1, 07747 Jena, Germany; 2 Strahlentherapie RheinMain, Rüsselsheim, Germany; 3 Klinik für Strahlentherapie und Radioonkologie, Franziskus Hospital, Bielefeld, Germany; 4 RNS Praxisgemeinschaft, Radiologie und Strahlentherapie, Wiesbaden, Germany; 5 Klinik für HNO-Erkrankungen, Kopf-Hals-ChirurgieInterdisziplinäre Palliativstation, Südharz Klinikum Nordhausen, Nordhausen, Germany; 6 Klinik für Hämatologie und Onkologie, Universitätsklinikum Göttingen, Göttingen, Germany

**Keywords:** Neoplasm, Cancer patients, Carcinogenesis, Lay etiology, Complementary medicine, Alternative medicine

## Abstract

**Background:**

The usage of complementary and alternative medicine (CAM) is widespread among cancer patients. While reasons for and aims of using CAM have been evaluated in many studies, less is known about whether patients’ concepts of how and why cancer develops has an influence on the choice of the CAM method.

**Methods:**

We pooled the data from all studies of our working group containing questions on lay etiological concepts and CAM usage and reanalyzed them with respect to the associations between these parameters.

**Results:**

The pooled dataset from 12 studies included 4792 patients. A third (1645 patients) reported using CAM. Most often used were supplements (55.9%), relaxation techniques (43.6%), and homeopathy (37.9%). Regarding perceived causes, patients most often marked stress (35.4%) followed by genes (31.9%). While all lay etiological beliefs were highly significantly associated with usage of CAM in general, there was no association between single lay etiological concepts and types of CAM used. Yet, in a network analysis, we found two associations: one comprising trauma, mistletoe, genes, and nutritional supplements, the other yoga, vitamin C, nutritional supplements, and TCM herbs. In the correlation heatmap, one cluster comprises etiological concepts of personality, immune system and trauma, and two clusters of CAM methods emerged: one comprising praying, yoga, meditation, and relaxation procedures, the other nutritional supplements, selenium, vitamins A and C.

**Conclusion:**

While physicians are trained to derive treatment strategies from etiological concepts, lay people choosing CAM do not follow these rules, which may point to other needs of patients addressed by CAM.

## Introduction

Cancer patients often use complementary or alternative medicine (CAM). Numbers in studies from western countries range between 30 and 90 percent, depending in part on type of cancer, gender and definition of CAM (Molassiotis et al. 2005; Micke et al. 2009; Zeller et al. 2013; Huebner et al. 2016; Loquai et al. 2017; Dubois et al. 2020; Bauer et al. 2018).

Reasons to visit naturopaths or non-medical practitioners are heterogeneous. Yet, one decisive argument is that both take time during the visits to listen and to discuss (Huebner et al. 2014a). While the treatment concepts of modern oncology are complicated and hard to understand for lay persons, the alleged mechanisms of CAM methods often address concepts of illness that are easier to understand. Examples are Homeopathy and the Simile Principle, which suggest that diseases are treated with preparations, which in a healthy person may evoke similar symptoms as those of the patient. In many traditional medicine systems, illness is a sign of disbalance between internal forces, and the healing comes from rearranging these forces—for example, the Qi in Traditional Chinese Medicine.

On the other hand, treatments like chemotherapy may not feel trustworthy if the patient believes that cancer has developed due to toxins getting into the body, since chemotherapy is often described as toxins against (tumor) cells.

In fact, lay persons have concepts of why they got cancer which may differ from modern scientific concepts. In our own studies, lay persons frequently suspected stress and mental trauma as the cause of cancer. Environmental pollutions and toxins are also often named (Huebner et al. 2014a, b). In contrast, most patients and relatives did not consider smoking, alcohol, or unhealthy diet as relevant.

Most often used CAM methods are micronutrients, herbs and mind–body techniques. Yet the field is highly heterogenous, also comprising the so-called holistic systems or energy healing. Aims of using CAM are also diverse (Huebner et al. 2014c). Most often, patients want to boost the immune system, increase one’s own strength, reduce side effects, gain control over the disease or receive a holistic treatment. Besides females and patients with higher education using CAM more often, not much is known on why some patients decide to use CAM while others do not (Huebner et al. 2014c, 2016). Moreover, data on why a patient chooses a special CAM method are missing.

If—from the patient’s point of view—conventional treatment methods are not in line with their etiological beliefs, one reason to look for CAM might be the attempt to find a method, which better fits one’s etiological beliefs.

Since 2011, we conducted several studies in diverse groups of cancer patients in different settings to learn more on CAM usage. In 12 of these studies, we also had a section asking for assumed causes of cancer (lay etiological concepts) (Huebner et al. 2016, 2014a, b, c; Loquai et al. 2017; Bauer et al. 2018; Dufter et al. 2021; Paul et al. 2013; Firkins et al. 2018; Hübner et al. 2022; Ebel et al. 2015; Kleine Wortmann et al. 2016; Halwas et al. 2017; Eisfeld et al. 2020; Welter et al. 2021; Hoppe et al. 2023; Ciarlo et al. 2021). So far, in these smaller studies, we did not find any associations. Therefore, we decided to reanalyze the pooled data.

## Methods and patients

### Patients

Participants were cancer patients during or after active cancer treatment with different types of cancer. Inclusion criteria were age above 18 years and the ability to fill in a questionnaire in German language. Exclusion criteria were patients not willing to participate or not being able to understand the questionnaire. Settings ranged from hospitals and ambulances to self-help groups and lectures for patients.

### Questionnaires

All questionnaires were standardized with closed questions. Considering lay etiology, we provided a list of items derived from the literature on the topic and added free lines. Patients were asked to mark one or several causes. For the question on CAM usage, in most surveys, we first asked the patients whether they used CAM at all and in case of yes offered a list of different CAM methods which we developed from a review of the literature in 2011 and adapted in small parts during the years as usage tended to vary a little bit (Huebner et al. 2014a).

The questionnaires were distributed in the respective settings in print or as online version using the German academic online survey tool “SoSci Survey” (https://soscisurvey.de).

### Ethical vote

All questionnaires were anonymous and the patients consented to participate by filling them in. For every survey, there was a positive vote of the ethical committee in charge.

### Statistics

We set up a separate SPSS data sheet (IMB Statistics SPSS Version 28) in which we first defined the variables overlapping in the different questionnaires. These were:

- Demographic data (gender, age, type of cancer; in some questionnaires, we also assessed education and religion)
- CAM usage (yes, no) and methods used (list with multiple choice)
- Causes of cancer (list with multiple choice)
- In some questionnaires, also aims of CAM usage (list with multiple choice)

From each SPSS data set or excel sheet of the single studies, we copied the respective data into the new data sheet. For this process, some variables’ coding had to be adapted (for example, coding of the types of cancer).

After that, the final dataset was analyzed using frequencies and Chi-Square tests for relations between demographic data or lay etiology and CAM usage. Effect sizes were calculated using Phi with φ < 0.2 (weak), φ = 0.2–0.6 (medium) φ > 0.6 (strong). For multiple testing, we used the Bonferroni method. *p* < 0.05 was considered statistically significant.

For both network analysis and correlation heatmap, the statistics module of the free online software ‘MetaboAnalyst’ (https://www.metaboanalyst.ca/) was used. Before using the dataset for health informatics analysis, however, we had to exclude 1000 cases from the initial sample size of 4192 due to missing information. A matrix of all data points was generated using an Excel spreadsheet. “Yes” answers were coded with a numerical value of “1”. Due to programming restrictions, “No” answers were coded with a numerical value of “0.0001”. If more than 50% of the values were missing, features were removed. Missing values were estimated as 1/5 of the minimum positive value of each variable. A network analysis was performed describing relations between lay etiologies and used CAM methods, as well as between etiology points itself or CAM methods itself (Chong et al. 2018).

The described correlation analyses were eventually used as base for a further principal component analysis. The resulting heat map (Heat Map Cluster Analysis) is able to illustrate the possible differences and similarities in relations among lay etiologies, used CAM rates, and between both parts.

## Results

### Demographic data

The pooled data from 12 studies included 4792 patients (Table 1) of whom 2481 (51.8%) were female and 1806 (37.7%) were male [504 missings (10.5%)]. Age ranged from 18 to 98 years with a mean of 66.1 years. Most frequent types of cancer were gastrointestinal cancer, including colorectal cancer (*N* = 1123; 23.4%), and melanoma (*N* = 1099 22.9%).

**Table 1:** Demographic data (*N* = 4792)

		*N*	%
Gender	Female	2034	42.4
	Male	692	14.4
	Missing	2066	43.1
Age	< 30 years	74	1.5
	31–50 years	692	14.4
	51–70 years	2034	42.4
	71–80 years	912	19.0
	> 80 years	200	4.2
	Missing	880	18.4
Education	4th class	825	17.2
	8–10th class	1604	33.5
	University entry diploma	834	17.4
	University diploma	588	12.3
	Missing	941	19.6
Type of cancer	Colorectal cancer	299	6.2
	Other gastrointestinal cancer	830	17.3
	Breast cancer	476	9.9
	Gynecological cancer	780	16.3
	Prostate cancer	220	4.6
	Other urogenital cancers	328	6.8
	Lung cancer	60	1.3
	Leukemia and lymphoma	321	6.7
	Melanoma	1099	22.9
	Others*	253	5.3
	Missing	126	2.6

^*^including non-melanoma skin cancer, head and neck cancer, glioma, sarcoma

### CAM usage

**Fig. 1:**
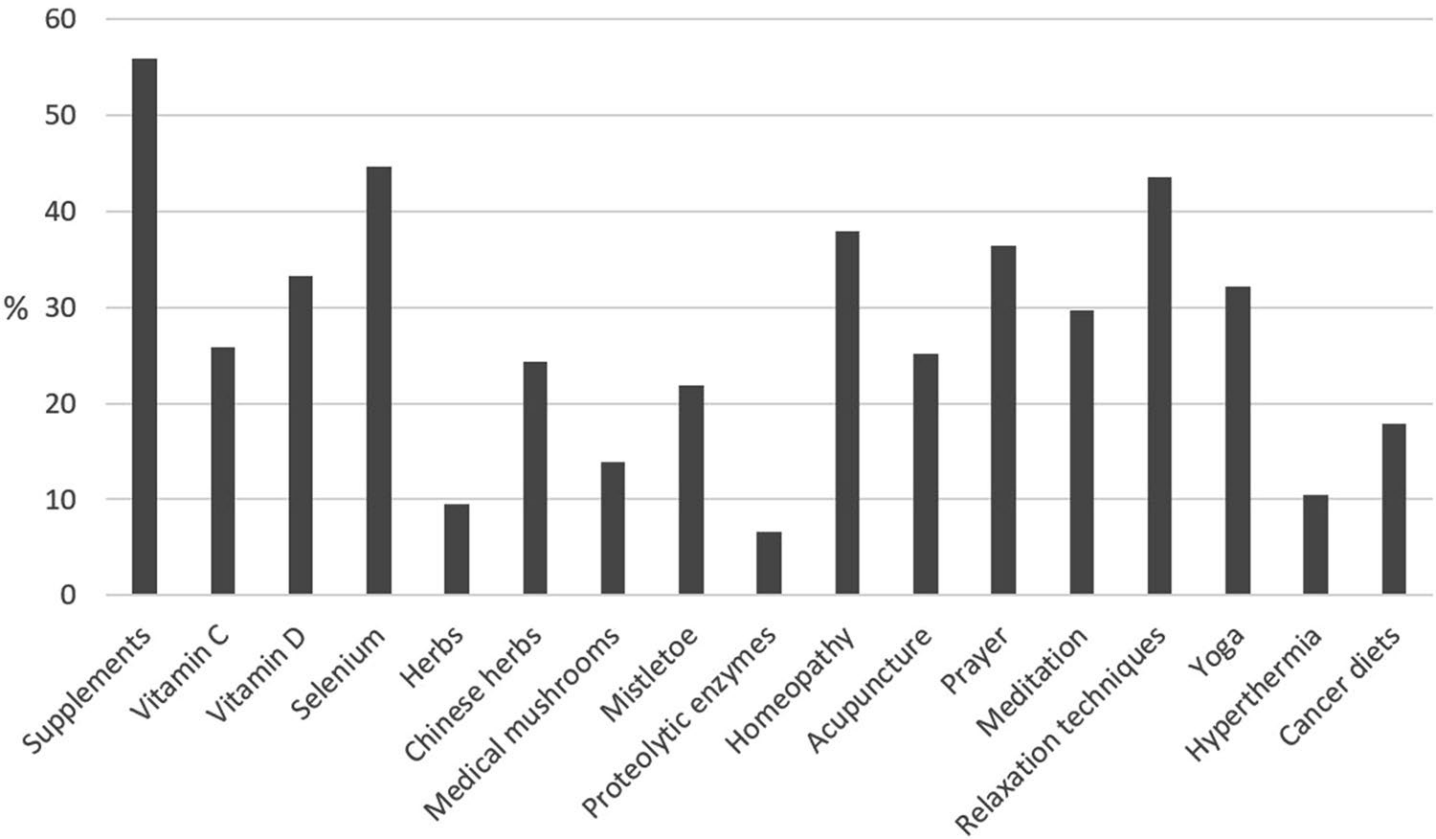
CAM methods used by the patients (*N* = 1645)

All in all, 1645 patients (34.3%) reported that they used CAM (Fig. 1). Most often used CAM methods were all types of supplements (*N* = 920; 55.9%), relaxation techniques (*N* = 718; 43.6%), homeopathy (*N* = 624; 37.9%) and prayer (*N* = 599; 36.4%). Among the supplements, most often named was selenium (*N* = 735; 44.7%).

Younger patients significantly more often used any CAM method (*V* = 0.223, *p* < 0.001), which was also true for the single methods listed. Female patients tended to use CAM more often but the effect was only weak (*V* = 0.103; *p* < 0.001). For education, there was a strong effect with higher educated patients significantly more often using CAM (*V* = 0.249; *p* < 0.001).

### Lay etiology

The questions on lay etiology were answered by 3445 patients. Most often, patients reported stress being the reason for falling ill with cancer (*N* = 1221; 35.4%) followed by genes (*N* = 1098; 31.9%) and unknown factors (*N* = 760; 22.1%). Lifestyle factors and smoking/alcohol were only named by a minority (*N* = 300; 8.7% and *N* = 358; 10.4% resp.).

Younger people more often named lifestyle (*V* = 0.398; *p* < 0.001), stress (*V* = 0.422; *p* < 0.001), environmental toxins (*V* = 0.403; *p* < 0.001) and genes (*V* = 0.403; *p* < 0.001). Men more often marked smoking and alcohol as cause (*V* = 0.361; *p* < 0.001). There was a strong association between higher education and believing that stress (*V* = 0.402; *p* < 0.001) or failure of the immune system (*V* = 0.251; *p* < 0.001) were the cause of cancer. In contrast, less educated people more often believed in mental trauma (*V* = 0.248; *p* < 0.001) and environmental toxins (*V* = 0.417; *p* < 0.001) as causes for cancer.

All lay etiological beliefs were highly significantly associated with usage of CAM in general. Table 2 presents the results of the Chi-Square tests for associations between lay etiology concepts and types of CAM used.

**Table 2:** Association between lay etiological belief and CAM methods used (φ*; N = 2456)

	Other lifestyle factors	Own personality	Stress	Trauma	Environmental toxins	Genes	Failure of immune system	Smoking/alcohol
Vitamin C	0.395	0.302	0.330	0.307	0.281	0.178	0.331	0.282
Vitamin D	0.481	n.s	0.403	0.208	0.425	0.404	0.202	0.475
Selenium	0.591	0.277	0.550	0.291	0.513	0.547	0.316	0.559
Herbs	0.482	0.321	0.451	0.352	0.443	0.466	0.343	0.479
Chinese herbs	0.629	0.373	0.545	0.397	0.556	0.574	0.415	0.598
Medical mushrooms	0.551	0.349	0.475	0.397	0.484	0.505	0.391	0.554
Mistletoe	0.666	0.357	0.606	0.403	0.606	0.628	0.396	0.661
Proteolytic enzymes	0.125	0.214	0.242	0.237	0.118	0.179	0.248	0.097
Homeopathy	0.570	0.378	0.536	0.412	0.516	0.538	0.407	0.564
Acupuncture	0.593	0.355	0.543	0.406	0.539	0.559	0.393	0.587
Prayer	0.582	0.380	0.541	0.413	0.520	0.533	0.408	0.564
Meditation	0.684	0.405	0.642	0.430	0.624	0.642	0.407	0.667
Relaxation techniques	0.586	0.403	0.548	0.433	0.524	0.535	0.419	0.551
Yoga	0.677	0.367	0.624	0.406	0.620	0.641	0.404	0.654
Hyperthermia	0.253	–	0.296	–	0.347	0.330	− 0.429	0.275
Cancer diets	0.533	n.s	0.442	0.210	0.499	0.480	0.194	0.536

^*^Color of the fields: white: φ < 0.2 (weak), gray: φ = 0.2–0.6 (medium), dark gray: φ > 0.6 (strong)

#### Network analysis

As shown in Fig. 1, there is only one strong positive association between etiological concepts and CAM methods: the concept of “alcohol and nicotine abuse” and “hyperthermia” occurred often. A medium association was seen for the couples “life style” and “hyperthermia”, “trauma” and “mistletoe” as well as “genes” and “nutritional supplements”. We have not documented associations between etiology concepts themselves. But there were strong positive associations between several CAM methods themselves—“yoga” and “vitamin C”, “nutritional supplements” and “TCM herbs”, “herbs” and “mushrooms”, “selenium” and “TCM herbs”. One negative interaction-couple was registered—“TCM herbs” and “vitamin C”.

#### Correlation heatmap

**Fig. 2:**
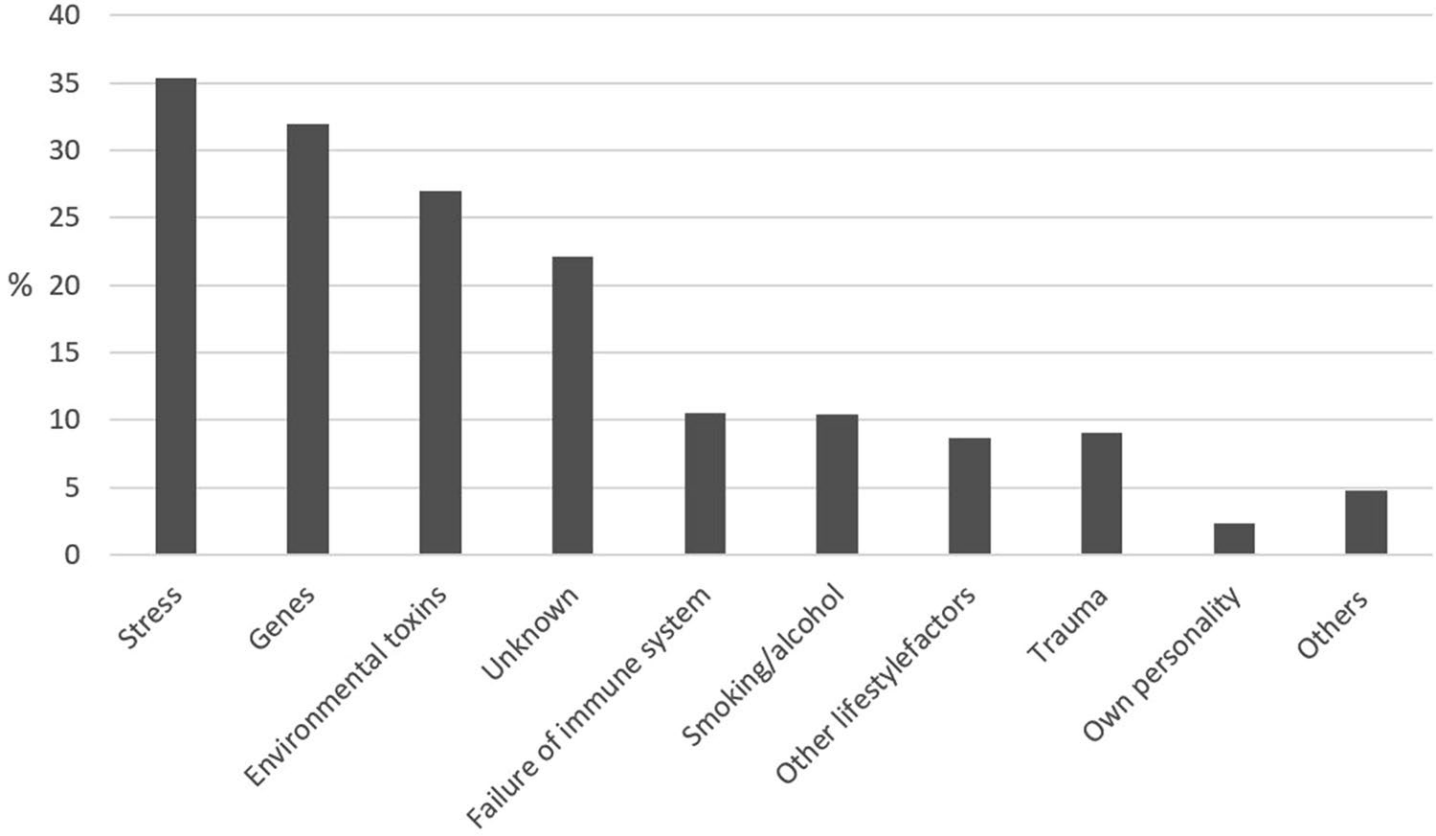
Causes of cancer from the patients’ point of view (*N* = 3445)

**Fig. 3:**
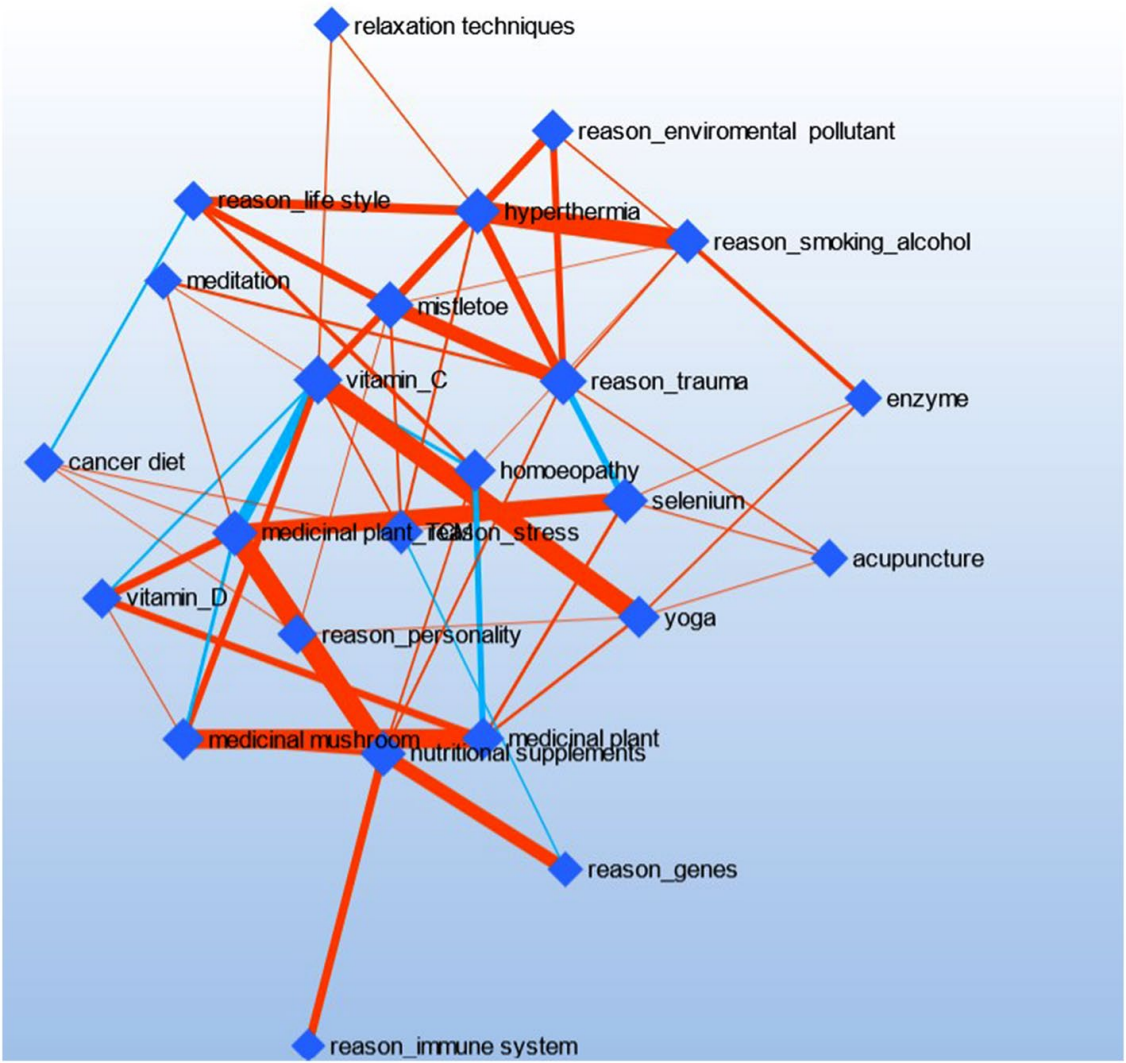
Network analysis of etiological concepts and CAM methods

**Fig. 4:**
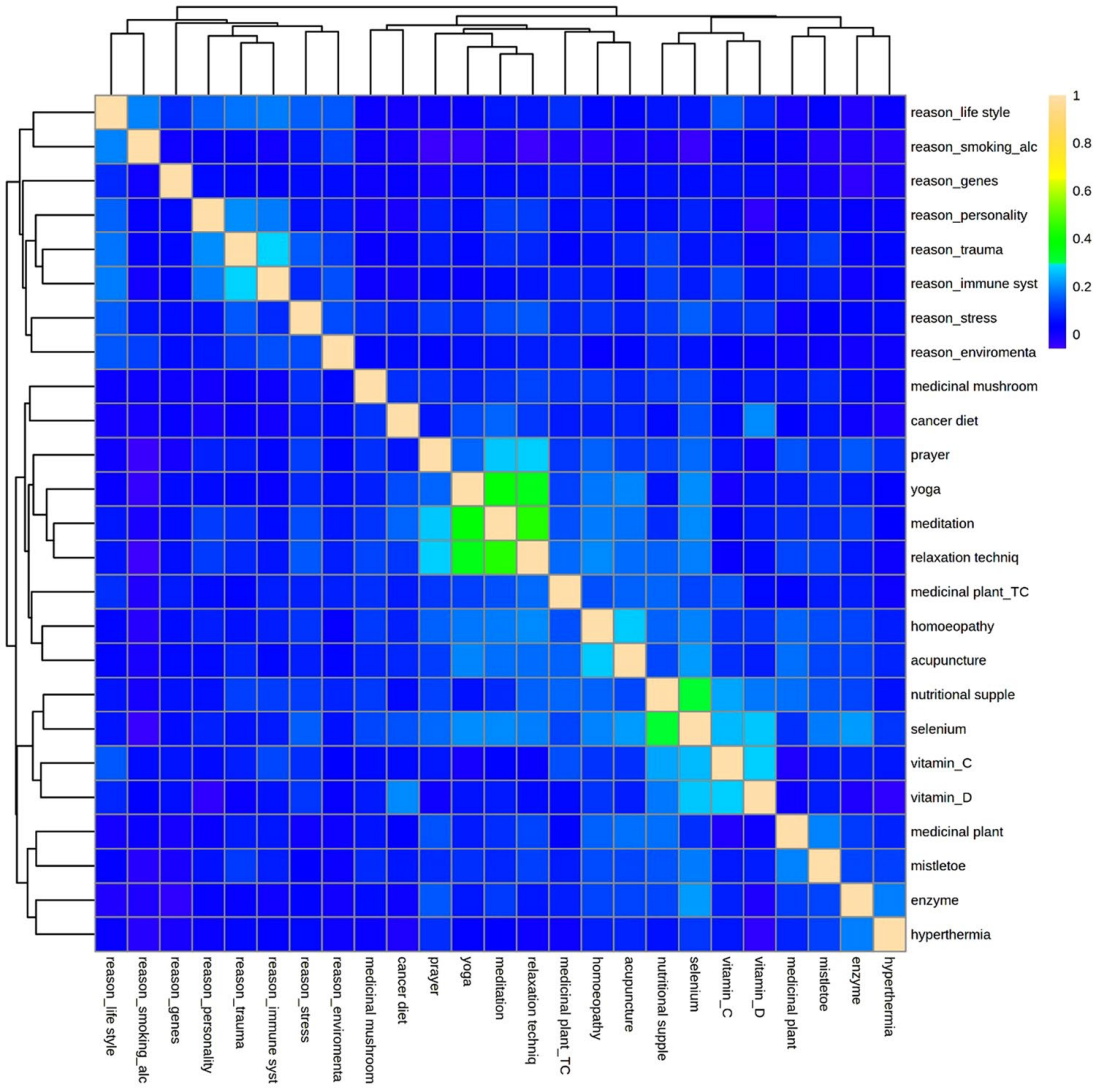
Correlation heatmap for relations between etiological and CAM methods

Fig. 2 demonstrates no strong relation between etiology concepts and the favored CAM methods,but there are some documented clusters. Etiology concepts of personality, immune system and trauma have built such a cluster. Praying, yoga, meditation, and relaxation procedures are parts of the first CAM cluster. The second CAM cluster contains nutritional supplements, selenium and the vitamins A and C (Figs. 3, 4).

## Discussion

About a third of participants reported CAM usage, which is a little bit below the cumulated data reported by other authors for Germany (Horneber et al. 2012). This may be explained by the large study on melanoma patients included in the data set, who reported a rather low user rate. Moreover, in the surveys including only patients living in the former eastern part of Germany, we found a lower rate as well. With respect to CAM methods use, micronutrients and other supplements were most often reported, followed by relaxation techniques, homeopathy and prayer. In line with the literature, younger patients, female patients and higher educated patients used CAM more often (Horneber et al. 2012).

The lay etiological concepts of the participants are in line with the data reported in literature (Molassiotis et al. 2005; Andersen et al. 2017). Most often, patients reported stress, followed by genes. Lifestyle factors and smoking/alcohol were only named by a minority. Genes were gaining importance in our surveys at a time when genetic predisposition was increasingly discussed in public by scientists and physicians as well as celebrities—most prominently Angelina Jolie. Younger people more often named lifestyle, stress, environmental toxins and genes. The strong association between high education and stress as the presumed cause of cancer might point to an experience of more stress by people in professions with higher responsibility and often more demanding time tables.

While all lay etiological beliefs were highly significantly associated with usage of CAM in general, we found no pattern in the analysis of favored CAM methods and lay etiology. In fact, patients do not seem to use a CAM-specific method, which might counteract or counterbalance an assumed cause of cancer. For example, meditation against stress seems to “make sense” but against other lifestyle factors as nutrition and lack of physical activity, this is counterintuitive. Moreover, also from the patient’s perspective, meditation most probably is no means against genes being the cause of a cancer disease. While the pattern in Table 2 for meditation and yoga is identical, relaxation techniques show a different pattern. On the other hand, the pattern of mistletoe is identical with that of meditation and yoga, suggesting that mistletoe—despite formally belonging to substance-based CAM—seems to fulfill rather psychological needs, which is in line with the anthroposophical concepts in which mistletoe plays a central part. This goes along with mistletoe showing strongest associations with mental trauma as presumed cause of cancer in the network analysis.

In the network analysis, there were strong associations between several CAM methods such as “yoga” and “vitamin C”, “nutritional supplements” and “TCM herbs”, “herbs” and “mushrooms”, “selenium” and “TCM herbs”. The latter all comprise substance-bound methods, which shows that one group of patients looking for additional help prefer to supplement medical treatment and conventional drugs with natural substances and drugs. The selection of those substances may depend on the available information for lay people and special advertising directed at cancer patients. In fact, the type of CAM methods used has been rather stable in the last decades. This cluster has also been emerged in our correlation heatmap. The second group of patients, as shown in the pattern of correlations as well as in the heatmap, uses methods from the cluster praying, yoga, meditation, and relaxation techniques, which may be characterized as mental practices. Both clusters may be characterized by a fit of expectations of the target patient/lay-person group and the respective presence in society that comes with commercial offers.

### Limitations

There are several limitations to our analysis. First of all, due to the pooling from different studies with slightly different questionnaires, for each analysis there is a substantial number of missings of demographic as well as CAM data. Moreover, the types of cancer are not representative as, for example, the largest study was a multicenter study with patients with skin cancer. A part of the questionnaires was collected during lectures on CAM so that the rate of CAM usage might be larger than in another setting. Yet, the rate of the whole collective is rather lower than reported from studies of other authors during the same time.

In our study, we have focused on lay etiology of cancer and CAM. We did not ask whether patients used CAM to cope with certain side effects of cancer treatment. Yet, we have done several studies on people’s reasons to look for CAM, and treating side effects was only named by a minority of the participants (Huebner et al. 2014a, 2016; b; Paul et al. 2013; Firkins et al. 2018).

## Conclusion

For the counseling of cancer patients, our results are important. There are some patients with a decisive medical need, like certain side effects from cancer treatment. After checking whether supportive care in these cases is comprehensive, complementary methods may be offered according to the clinical evidence, weighing benefits and risks (Onkologie and (Deutsche Krebsgesellschaft, Deutsche Krebshilfe,_AWMF) 2021). For these patients, an evidence-based nutrition counseling pointing to the content of micronutrients in a well-balanced diet may be helpful. The German S3 guideline additionally recommends measuring vitamins B12, D and selenium (Onkologie and (Deutsche Krebsgesellschaft, Deutsche Krebshilfe,AWMF) 2021). Both, nutrition counseling and defined analysis with supplementation in case of deficits may easily be integrated in comprehensive cancer care.

A large group of patients has a more general need for becoming active, supporting their own physical and mental strength to better cope with the disease and its treatment. These patients may choose their favorite techniques according to their own preferences and the offers in the region they live in. Physicians may also use this interest to point to the high importance of physical activity and sports.

In a former study, we have shown that CAM usage is associated with a high external locus of control and not at all with self-efficacy (Ebel et al. 2015). While counseling patients asking for CAM, it might be helpful to also include explanations on carcinogenesis and to explain what may be achieved by cancer treatments and what may be the realistic aims of integrative concepts, including nutrition, physical activity, complementary methods and spirituality.
